# Correlation Between Horizontal and Vertical Skeletal Components in Dental Malocclusions Among the Jazan Population

**DOI:** 10.7759/cureus.48087

**Published:** 2023-11-01

**Authors:** Wael Awadh

**Affiliations:** 1 Division of Orthodontics, Department of Preventive Dental Sciences, College of Dentistry, Jazan University, Jazan, SAU

**Keywords:** anterior facial height, skeletal malocclusion, dental malocclusion, horizontal and vertical components, posterior facial height

## Abstract

Introduction: The dentoskeletal morphology of various malocclusions has been analyzed in cephalometric studies. It is important to understand the vertical and horizontal components of orthodontic treatment. To provide accurate treatment, an orthodontist needs to understand the facial types of an individual. This study aims to evaluate a correlation between vertical and horizontal components of skeletal and dental malocclusion by assessing cephalometric radiographs of the population of Jazan province.

Methods: The cephalometric radiographs of 267 eligible participants were assessed digitally. Fourteen skeletal and dental parameters were used to evaluate the association. Reliability was checked with the intra-class coefficient. Statistical analyses included descriptive statistics and Spearman's rho test. Statistical significance was set at P ≤ 0.05.

Results: Correlations were found between anterior facial height (AFH), posterior facial height (PFH), FH ratio (Jarabak ratio), upper incisor to NA (U1-NA), lower incisor to NB (L1-NB), and upper incisor to the palatal plane (U1/PP). In dental class I, AFH (N-Me) had a strong positive correlation with L1-NB (0.300), U1/PP (0.164), and L1/MP (0.215). In dental class II, AFH negatively correlated with U1-NA (−0.735) and positively correlated with L1-NB (0.292), L1/MP (0.085), and U1-NA. PFH (S-Go) positively correlated with L1-NB (0.525) in class I but negatively correlated in class II. However, a negative relation was observed between all the vertical and horizontal components in class III.

Conclusion: This study suggests potential associations between vertical and horizontal components in developing skeletal and dental discrepancies.

## Introduction

Dental and skeletal morphology have been examined in several cephalometric investigations. Planning dentofacial orthodontic treatment for malocclusion depends on understanding the facial and skeletal anatomy. The dental profession is highly demanded to address the consequences of craniofacial and occlusal anomalies [1]. Orthodontics and surgery are sometimes used to address malocclusion and boost a person's self-esteem [2]. There is a lack of tools in the field that can assist in the early detection of a growth pattern that may result in detrimental craniofacial and occlusal relationships [3]. Hence, it is essential to thoroughly evaluate the attributes of horizontal and vertical relations to establish the treatment objective.

Various cohort and cross-sectional studies have measured malocclusions' vertical and horizontal growth patterns [4-7]. A comparative study by Riesmeijer et al. [8] demonstrated that dental class II subjects had greater Sella-Nasion-point A (SNA) and SN-GoMe angles than class I. Chung and Wong concluded that SN-mandibular plane angle affected craniofacial growth in skeletal and dental Class II subjects aged 9 to 18, with low, medium, and high angles [9]. At age 9, the high-angle group had greater convexity, larger Y-axis and gonial angles, and greater anterior facial height (AFH) [9,10]. However, at 18, all groups had decreased facial convexity and increased mandibular forward rotation [9]. Conversely, a Syrian study investigator reported smaller vertical facial dimensions and shorter anterior facial height with classes II and III malocclusion [11]. The prevalence of dental class I is reportedly high in the northern province of Saudi Arabia [3]. Moreover, the compensatory mechanism in skeletal and dental structures is recorded to maintain a balanced and proportional facial pattern when deviations in growth patterns result in various malocclusions. In various studies, dental and skeletal malocclusions are studied separately, even though studies on the growth patterns of the maxilla and mandible emphasize the importance of vertical growth and its correlation to anteroposterior growth [7,12].

A few researchers have focused on the correlation between vertical and horizontal growth patterns. However, measuring facial growth patterns without a stable reference plane is challenging. ANB and functional occlusal plane angles initially measured the anteroposterior maxillary and mandibular plane relationship [13], which was further popularized as "Witts appraisal" [14]. Moreover, Nanda and Merril [15] employed the palatal plane to calculate the perpendicular projection from points A and B to represent the anterior maxillary and mandibular relationship [15]. Numerous authors have pointed out the errors in these analyses; for instance, the ANB angle is affected by the position of the nasion point and jaw rotations [16], the Wits appraisal is affected by the occlusal plane and the vertical growth of the maxilla and mandible [15], and the A-B points could be affected by the palatal plane rotation [17]. Therefore, the relationship between the vertical and horizontal growth patterns is valid if the result of these patterns is like the previously defined classification of malocclusion [10]. Furthermore, studies have evaluated craniofacial and dental growth patterns separately. However, no studies have evaluated the importance of cephalometric landmarks and their relationship to developing skeletal and dental malocclusion. Hence, this retrospective study aims to evaluate a correlation between vertical and horizontal components of various malocclusions by assessing cephalometric radiographs of the population of Jazan province.

## Materials and methods

The protocol for this cross-sectional retrospective study was designed, and ethical approval was obtained from the Internal Committee of Research Unit, College of Dentistry, Jazan University, Kingdom of Saudi Arabia (Ref No. REC-45/03/762). The preliminary search consists of 350 adults seeking orthodontic treatment at the Orthodontic Clinical Department at the College of Dentistry as of January 2017. The research was conducted following the principles of the Declaration of Helsinki. The inclusion criteria for participants are as follows: (1) age greater than or equal to 17 years; (2) absence of hereditary diseases or syndromes impacting the craniofacial region; (3) no prior orthodontic treatment; and (4) no significant craniofacial surgeries. Finally, a total of 267 participants were recruited for this study. All patients provided electronic informed consent before registering in the institute's database, granting permission to use their data for research purposes.

The lateral cephalometry of those meeting the criteria was analyzed using automated digital software (Morpheus, Korea). The Frankfort horizontal plane was aligned parallel to the floor during imaging. Patients were advised to keep their bite in maximum intercuspation through cephalometric imaging to prevent inaccurate vertical and horizontal skeletal findings. Cephalometric radiographs were obtained using a Rotograph Plus model MR05 radiographic unit, with the settings adjusted to 85 kilovolts peak (kVp), 10 milliamperes (mA), and an exposure time of 0.5 seconds. The radiographic equipment remained stable and consistent, with a fixed focus-object distance of 1.5 m. Kodak TMG/RA film measuring 20.3 cm × 25.4 cm was positioned at 15 cm from the patient's mid-sagittal plane, resulting in an approximate magnification of 10%.

The number of samples was calculated using G Power software from the University of Düsseldorf (Düsseldorf, Germany). A significance level of 0.05 and a statistical power of 90% were utilized. The power analysis determined that a sample size of 15 participants in each skeletal class and 51 participants in each facial pattern was required. The analysis employed was based on the data from previous studies. Freudenthaler et al. [7] found that the inclination of mandibular incisors to the MP line was 94±10° for class II malocclusions and 82.5±5.8° for class III malocclusions. Modi et al. [16] determined that the position of mandibular incisors to the NB line was 31.45±7.69 mm for individuals with a hyper-divergent face and 26.15±6.62 mm for those with a hypo-divergent facial pattern.

**Figure 1:**
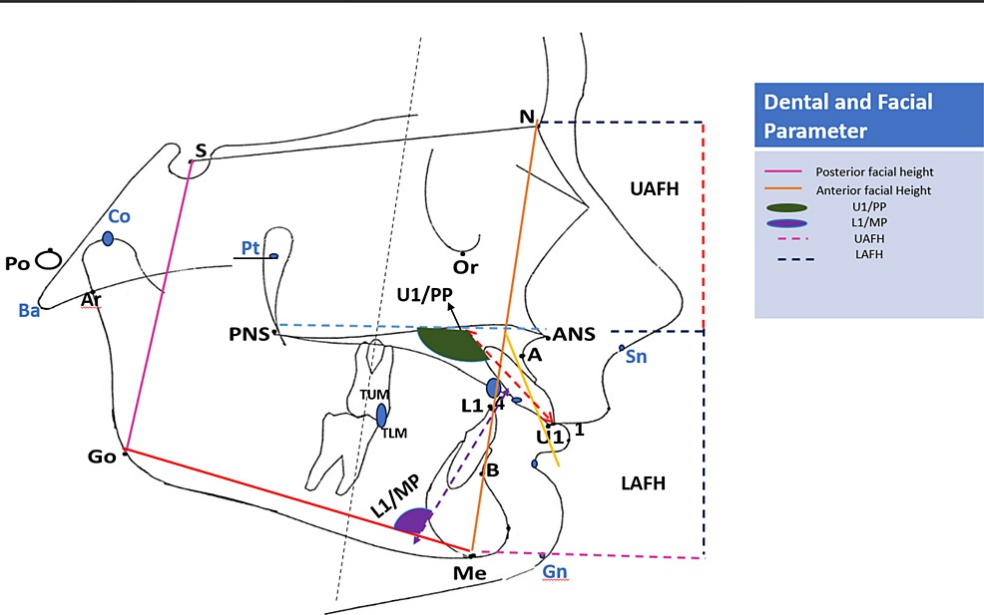
Dental and skeletal parameters measured in this study (1) U1PP: maxillary incisors to palatal plane; (2) L1/MP: lower incisors to mandibular plan; (3) UAFH: upper anterior facial height; (4) LAFH: lower anterior facial height (This figure was created by the author [Dr. Wael Awadh]).

The study included 267 participants, with 134 males and 138 females. From the total sample, 131 participants were skeletal class I (ANB 1-3°), 83 were skeletal class II (ANB >4°), and 53 were skeletal class III (ANB <0°). For dental malocclusion, a total of 195 were class I, 51 were class II, and 21 were class III patients. Patients were categorized as dental class I if they exhibited anterior crowding but had a normal molar relationship. For dental class II, the disto-buccal cusps of the maxillary molar articulate with the mesial to buccal grooves of the mandibular first molar. Dental class III patients had a posteriorly positioned maxillary molar in relation to the mandibular first molar. The dental class malocclusion (class I, II, and III) was mentioned in patients' record files. All participants were aged 17 and above, had complete permanent dentition, and had no history of orofacial malformation or surgery. The ANB angle and A-B difference to Nasion vertical (mm) were measured to categorize patients into skeletal malocclusion. Lateral cephalometric analysis was started, utilizing various anatomical landmarks for dental and skeletal malocclusion (Figure 1 and Table 1).

**Table 1:** Cephalometric parameters utilized in the study *Dental parameters:* U1-NA: upper incisor to nasolabial angle (NA), U1-NB: upper incisor to nasal base (NB), L1-NB: lower incisor to NB, U1/PP: angle between midline of upper incisor with a palatal plane, L1-MP: lower incisor to mandibular plane. *Skeletal parameters: *SNA: sella-nasion-point A, SNB: sella-nasion-point B, Pog N perp: pogonion to nasion perpendicular, ANB: Abdiff (NV): the linear difference between A-NV and B-NV; A-NV: the linear difference between point A and nasion, B-NV: the linear distance between point B and nasion, AFH: anterior facial height, PFH: posterior facial height, UAFH: upper anterior facial height, JR: Jarabaks ratio, LAFH: lower anterior facial height.

Dental parameters	U1-NA
	U1-NB
	L1-NA
	L1-NB
	U1/PP
	L1-MP
Skeletal parameters	SNA
	SNB
	ANB
	Ab diff (NV)
	PFH (S-Go)
	UAFH (N-ANS)
	JR (S-Go/N-Me)(FH ratio)
	LAFH (ANS-Me)

Statistical analysis

All the data were analyzed utilizing SPSS version 21.1 (IBM, Chicago, IL, USA). Fifteen lateral cephalometric radiographs were randomly selected and analyzed independently to check the consistency of the findings. The reliability of the measurement was assessed by utilizing the intra-class correlation coefficient (ICC). Descriptive statistics were employed to calculate the mean, median, and standard deviation (minimum and maximum). Non-parametric Spearman-Rho tests were used to determine the possible correlation between dental and skeletal malocclusion. The value was significant at P≤0.05.

## Results

The intra-class correlation coefficients ranged from 0.923 to 0.988 (p < 0.01) for all measurements. This finding supports the reliability of the investigator's repeated measurements. The mean and standard deviation of each parameter are described in Table 2. The correlation test utilized in this study is Pearson correlation, with a statistical significance of P ≤ 0.05. Table 6 illustrates a negative correlation between the measurement of facial heights (AFH, PFH, UAFH, LAFH, and FH ratio) and maxillary mandibular relations. Additionally, a weak relationship is measured between facial proportion and dental measurements. Notably, anterior facial height, posterior facial height (PFH), Jarabak ratio (FH ratio), and F proportion consistently exhibit positive associations with horizontal components. Conversely, different dental classes (PFH = −0.050; UAFH = −0.028; FH ratio = −0.101) and U1/PP (AFH = −0.069; PFH = −0.046; UAFH = −0.110; FH ratio = −0.020) showed a negative or weak association with almost all the measurements (Table 7). Table 8 demonstrates the correlation between dental class and the positive association between U1-NA (0.044) and U1/PP (0.046). At the same time, no significant relationship was found between other dental angles (U1-NA and L1/MP; L1-NB and L1/MP; U1/PP and L1/PP).

The correlation between skeletal and dental classes demonstrates a strong positive relationship between A-NV and the L1-NB angle and a negative relationship between A-NV and the U1-NA angle. In contrast, no relation is demonstrated between A-NV and U1/PP or L1/MP angles. Conversely, a strong positive relationship is established between B-NV and U1-NA and B-NV and U1/PP. However, no correlation was measured between L1/MP (Table 9).

**Table 2:** Descriptive data for measurement of variables

	Mean±SD	Minimum	Maximum
AFH (N-Me)	125.2±7.4	107.0	147.5
PFH (S-Go)	79.3±7.2	63.4	99.0
UAFH (N-ANS)	55.4±3.3	48.3	65.0
LAFH (ANS-Me)	69.7±5.9	56.0	85.0
FH ratio SGo/Nme	63.1±4.3	50.0	76.0
F prop LAFH/Nme	55.4±2.3	50.0	61.0
A-NV	−0.8±3.3	−13.0	7.0
B-NV	−5.4±5.1	−19.0	9.7
Ab diff (NV)	4.6±3.8	−5.3	13.1
SNA	82.1±3.2	72.0	89.0
SNB	79.8±2.9	73.0	89.0
ANB	2.4±2.6	−7.0	7.6
U1–NA	5.8±3.3	−3.5	16.0
Dental class	6.8±2.6	0.0	16.8
U1/PP	113.1±8.1	86.8	137.5
L1/MP	96.3±7.4	75.0	114.5

Table 3 illustrates that a non-parametric test for correlation shows no or weakly significant relationship between various horizontal angles and the inclination of mandibular teeth. The parametric Pearson test measured a positive association between dental class I angles (A-NV, B-NV, and SNA) and mandibular tooth inclination (Table 6). However, no correlation was found between AB diff and ANB with mandibular tooth inclination for all the dental classes.

The result of the non-parametric spearmen rank test illustrates a weak relation between dental class I and U1-NA (−0.252**) and a strong relation between other angles of dental class I and L1-NB (0.300**) and L1/MP (0.215) moderate with U1/PP (0.164). However, a negative association was reported with Ab diff and ANB in class I with all the angles of mandibular incisors. Moreover, the ANB angle possesses a weak association with mandibular incisors in dental class I. The association of angles in dental class II was reported with SNA and U1/PP (0.164*). A negative relationship was measured between dental class II and various parameters (A-NV and U1-NA-0.325**, B-NV and L1NB-0.159) (Table 3). Similarly, the ANB angle in dental class II measures a negative association with U1-NA (−0.735) and U1/PP (−0.460). In dental class III, horizontal and vertical measurements show a positive correlation between SNB and U1/PP (0.913). On the contrary, ANB in dental class III shows a strong negative correlation between U1-NA and U1/PP. In comparison, all the other angles measure a strong negative or no significant correlation (Table 3).

**Table 3:** Correlation between different horizontal angles and teeth inclination of each dental class P-value < 0.05 **Spearmen correlation coefficient: no correlation (0.0<0.01) low correlation (0.1<0.3) moderate correlation (0.3<0.5) high (0.7)

Dental class			U1–NA	L1-NB	U1/PP	L1/MP
Class I	A-NV	rho	−0.252^**^	0.300^**^	0.164^*^	0.215^**^
		P	0.000	0.000	0.022	0.003
	B-NV	rho	0.138	−0.036	0.470^**^	0.001
		P	0.055	0.622	0.000	0.993
	Ab diff (NV)	rho	−0.468^**^	0.350^**^	−0.507^**^	0.215^**^
		P	0.000	0.000	0.000	0.003
	SNA	rho	−0.225^**^	0.302^**^	0.144^*^	0.179^*^
		P	0.002	0.000	0.045	0.012
	SNB	rho	0.181^*^	−0.044	0.463^**^	−0.046
		P	0.011	0.543	0.000	0.527
	ANB	rho	−0.582^**^	0.449^**^	−0.381^**^	0.292^**^
		P	0.000	0.000	0.000	0.000
Class II	A-NV	rho	−0.324^*^	0.076	−0.030	0.070
		P	0.020	0.596	0.832	0.626
	B-NV	rho	0.268	−0.159	0.329^*^	0.080
		P	0.058	0.264	0.019	0.574
	Ab diff (NV)	rho	−0.624^**^	0.298^*^	−0.535^**^	−0.033
		P	0.000	0.034	0.000	0.816
	SNA	rho	−0.251	0.023	−0.155	0.007
		P	0.075	0.873	0.279	0.959
	SNB	rho	0.449^**^	0.000	0.521^**^	0.183
		P	0.001	0.999	0.000	0.198
	ANB	rho	−0.735^**^	0.255	−0.460^**^	0.117
		P	0.000	0.071	0.001	0.412
Class III	A-NV	rho	−0.484^*^	0.142	0.067	0.408
		P	0.026	0.539	0.772	0.066
	B-NV	rho	0.073	−0.376	0.230	−0.186
		P	0.752	0.093	0.316	0.420
	Ab diff (NV)	rho	−0.402	0.731^**^	−0.124	0.610^**^
		P	0.071	0.000	0.592	0.003
	SNA	rho	−0.491^*^	0.134	0.070	0.392
		P	0.024	0.562	0.763	0.079
	SNB	rho	0.026	−0.324	0.260	−0.109
		P	0.913	0.152	0.255	0.639
	ANB	rho	−0.678^**^	0.642^**^	−0.022	0.706^**^
		P	0.001	0.002	0.926	0.000

Table 4 illustrates the association of vertical angle with mandibular tooth inclination, resulting in a strong positive correlation of AFH (N-Me) with U1-NA (0.233) and L1-NB (0.292) in class I. Moreover, statistical significance was recorded between AFH and U1-NA (0.001). In class II, AFH had a negative and weak association with U1-NA (−0.020) and U1/PP (−0.080). Conversely, AFH in dental class III showed a strong correlation and statistical significance with U1-NA (0.608) and a negative and weak correlation with L1/MP (−0.206). Similarly, PFH (S-Go) shows a strong correlation with U1-NA (0.155) and a weak relationship with U1/PP (−0.089) in dental class I. Conversely, in class II, PFH measured a weak and negative correlation with U1/PP and U1-NA, and with other angles, PFH in class II also measured a moderate correlation (Table 4). In class III, PFH measures a strong correlation with U1-NA (0.543) and no or weak association with other angles. In comparison, UAFH measured a strong negative correlation in classes I and II with U1-NA (−0.046 and −0.397), U1/PP (−0.089 and −0.310), and L1/MP (−0.181 and −0.148). Conversely, with dental class III, UAFH strongly correlates positively with U1-NA (0.412) and U1/PP (0.334). LAFH in classes I, II, and III measures a strong positive correlation with U1-NA (0.305, 0.179, and 0.533) and L1-NB (0.392, 0.085, and 0.086). A negative or weak relationship was measured between other angles and LAFH in all three dental malocclusions. The FH ratio measures a strong relationship with L1/MP (0.255 and 0.525) in classes I and II and a negative or weak association with L1-NB (−0.161). Similarly, F prop measures a strong relationship with U1-NA (0.278 and 0.406) in dental malocclusion classes I and II; no association was recorded with class III.

**Table 4:** Correlation between vertical angles and anterior teeth position/inclination of each dental class P-value < 0.05 **Spearmen correlation coefficient: no correlation (0.0<0.01) low correlation (0.1<0.3) moderate correlation (0.3<0.5) high (0.7)

		Class I (N= 195)				Class II (N= 51)				Class III (N= 21)			
		U1–NA	L1-NB	U1/PP	L1/MP	U1–NA	L1-NB	U1/PP	L1/MP	U1–NA	L1-NB	U1/PP	L1/MP
AFH (N-Me)	Rho	0.233^**^	0.292^**^	−0.117	−0.127	−0.020	0.072	−0.081	−0.217	0.608^**^	0.120	0.267	−0.206
	P	0.001	0.000	0.104	0.077	0.888	0.617	0.574	0.126	0.003	0.604	0.242	0.370
PFH (S-Go)	Rho	0.155^*^	0.024	−0.089	0.067	-0.034	0.110	−0.095	0.213	0.543^*^	−0.145	0.329	−0.103
	P	0.031	0.738	0.217	0.353	0.814	0.441	0.509	0.133	0.011	0.532	0.146	0.658
UAFH (N-ANS)	Rho	−0.046	−0.081	−0.098	−0.181^*^	−0.397^**^	0.012	−0.310^*^	−0.148	0.452^*^	−0.269	0.334	−0.328
	P	0.526	0.263	0.174	0.011	0.004	0.936	0.027	0.300	0.040	0.238	0.140	0.146
LAFH (ANS-Me)	Rho	0.305^**^	0.392^**^	−0.116	−0.067	0.179	0.085	0.053	−0.187	0.533^*^	0.086	0.078	−0.187
	P	0.000	0.000	0.106	0.351	0.210	0.552	0.712	0.188	0.013	0.712	0.736	0.416
FH ratio SGo/Nme	Rho	0.031	−0.161^*^	−0.037	0.255^**^	−0.051	0.233	−0.063	0.525^**^	0.125	0.035	0.334	0.260
	P	0.665	0.025	0.606	0.000	0.722	0.101	0.661	0.000	0.588	0.881	0.139	0.254
F prop LAFH/Nme	Rho	0.278^**^	0.391^**^	−0.090	0.016	0.406^**^	0.075	0.162	−0.112	0.155	0.342	0.020	0.091
	P	0.000	0.000	0.213	0.820	0.003	0.601	0.257	0.433	0.502	0.130	0.932	0.695

Table 5 shows that AFH (N-Me) is negatively correlated with A-NV (−0.252), B-NV (−0.327), and positively correlated with Ab diff (NV) (0.253) in class I. In class II, AFH negatively correlates with A-NV (−0.099), B-NV (−0.179), and SNB (−0.113). In class III, AFH negatively correlates with A-NV, B-NV, Ab diff (NV), SNA, and ANB (−0.291), while positively correlates with SNB angle. On the contrary, PFH (S-Go) negatively correlates with A-NV, B-NV, and SNB in class I. While in class II, PFH negatively correlates with B-NV (−0.220), Ab diff (NV), and ANB (0.013). Similarly, UAFH (N-ANS) negatively correlates with A-NV, B-NV, and SNB. In class II, UAFH negatively correlates with B-NV and Ab diff (NV) and positively correlates with SNB. In class III, UAFH positively correlates with Ab diff (NV) and negatively correlates with SNB (Table 5). LAFH measured a strong negative relationship with A-NV (−0.238), B-NV (−0.337), SNA (−0.200), and SNB (−0.293) and a positive relationship with ANB (0.233) in skeletal class I. While there is a weak or negative association between LAFH and A-NV, B-NV, SNA, SNB, and ANB in class II, conversely, LAFH measured a strong negative relationship with ANB (−0.352) in skeletal class III. The FH ratio showed a strong positive association with SNA and SNB in class I, while there was a negative correlation with ANB (−0.013). In class II, the Jarabak ratio is strongly associated with Ab diff, while a weak association was recorded with A-NV (−0.262) and SNA (−0.263). However, a negative relationship was observed between all the vertical and horizontal components in class III.

**Table 5:** Correlation between vertical and horizontal skeletal measurement of each dental class P-value < 0.05 **Spearmen correlation coefficient: no correlation (0.0<0.01), low correlation (0.1<0.3), moderate correlation (0.3<0.5), high (0.7)

		Class I (N= 131)						Class II (N= 83)							Class III (N= 53)					
		A-NV	B-NV	Ab diff (NV)	SNA	SNB	ANB	A-NV	B-NV		Ab diff (NV)	SNA	SNB	ANB	A-NV	B-NV	Ab diff (NV)	SNA	SNB	ANB
AFH (N-Me)	rho	−0.252^**^	−0.327^**^	0.283^**^	−0.210^*^	−0.279^**^	0.162	−0.099		−0.179	0.160	−0.073	−0.113	−0.054	−0.230	−0.098	−0.006	−0.125	0.053	−0.291^*^
	P	0.004	0.000	0.001	0.016	0.001	0.064	0.372		0.106	0.150	0.512	0.308	0.626	0.098	0.484	0.968	0.371	0.709	0.034
PFH (S-Go)	rho	−0.059	−0.091	0.071	−0.034	−0.070	0.085	−0.178		−0.220^*^	0.104	−0.183	−0.201	−0.103	−0.130	−0.009	−0.033	−0.077	0.094	−0.237
	P	0.501	0.300	0.419	0.696	0.427	0.334	0.108		0.046	0.348	0.097	0.068	0.354	0.352	0.949	0.813	0.584	0.503	0.087
UAFH (N-ANS)	rho	−0.095	−0.108	0.070	−0.087	−0.105	0.000	−0.085		−0.226^*^	0.324^**^	−0.104	−0.155	0.208	−0.100	0.042	−0.060	0.018	0.168	−0.195
	P	0.279	0.220	0.425	0.322	0.234	1.000	0.447		0.040	0.003	0.349	0.162	0.060	0.476	0.766	0.672	0.896	0.228	0.163
LAFH (ANS-Me)	rho	−0.238^**^	−0.337^**^	0.325^**^	−0.200^*^	v0.291^**^	0.223^*^	−0.085		−0.097	0.002	−0.033	v0.066	−0.206	−0.248	−0.125	−0.024	−0.197	−0.006	−0.352^**^
	P	0.006	0.000	0.000	0.022	0.001	0.010	0.443		0.381	0.984	0.768	0.551	0.062	0.073	0.372	0.862	0.158	0.968	0.010
FH ratio SGo/Nme	rho	0.143	0.160	−0.144	0.156	0.165	−0.013	−0.216^*^		−0.202	0.028	−0.263^*^	−0.208	−0.160	0.029	0.031	0.056	0.002	0.022	0.028
	P	0.104	0.068	0.100	0.075	0.060	0.885	0.049		0.067	0.800	0.016	0.059	0.148	0.836	0.827	0.690	0.989	0.875	0.845
F prop LAFH/Nme	rho	−0.130	-0.221^*^	0.247^**^	−0.121	−0.200^*^	0.204^*^	−0.014		0.065	−0.201	0.027	0.056	−0.314^**^	−0.135	−0.084	−0.048	−0.172	−0.066	−0.214
	P	0.139	0.011	0.004	0.168	0.022	0.019	0.899		0.557	0.069	0.806	0.615	0.004	0.335	0.550	0.730	0.219	0.638	0.123

## Discussion

Subjects aged 17 and above were selected in this study for maximum craniofacial growth, and the skeletal and dental parameters are constant [8,9]. Numerous investigations have evaluated the skeletal and dental variations within the normal parameters [18-20]. Solow [21] proposed that the dentoalveolar compensatory mechanism is essential for coordinating tooth position with the underlying basal bones [21]. Along with stability, this mechanism is crucial for effective occlusion. The dentoalveolar compensating mechanism plays a significant role in providing excellent occlusion because the alignment of the upper and lower jawbones is not precise during and after growing phases, even in people with normal occlusion [21]. Similarly, Kim et al. recorded highly diverse skeletal decompensation in individuals with normal occlusion [22]. The results of these studies suggest the need for an understanding of the characteristics of dentoalveolar compensation to plan orthodontic treatment.

Adult patients with skeletal and dental discrepancies can be treated with functional appliances, orthodontic camouflage, or orthognathic surgery, requiring dentoalveolar compensation or decompensation for excellent treatment outcomes [5,6]. Anterior dentoalveolar compensation is essential in achieving successful orthodontic treatment, whether through orthodontic compensation or skeletal base correction (decompensation) [22].

In the present retrospective study, correlation analysis evaluated horizontal and vertical discrepancies. A negative correlation was recorded between ANB angle and maxillary incisor position in the case of a horizontal discrepancy. The increase in ANB angle correlates with retruded and retroclined maxillary incisors. Conversely, a positive correlation was obtained between the ANB angle (degree of anterior-posterior discrepancy) and mandibular incisors, resulting in protrusion and proclined mandibular incisors in relation to basal molar relations. These findings coincide with a study by Ishikawa et al. [23], who reported a positive correlation between ANB plane angle and mandibular incisors. Conversely, Ceylan and Eröz [24] recorded a positive relationship between maxillary incisors and sagittal angle and no statistical relation with mandibular incisors [24]. These differences among the studies could be attributed to the measurement of sagittal angles. Both studies measured overjet, while our study measured skeletal correlative parameters.

Our study recorded a significant positive correlation between dental classes and facial parameters. Notably, the FH ratio and F proportion measured positive associations with maxillary and mandibular jaw relations. Similarly, in the study by Alhammadi [25], a positive correlation was measured between posterior facial height and the mandibular incisor plane. In the study on the comparison of gender, it was recorded that females have greater posterior facial height and L1/MP angle than males [26]. Conversely, in the study on the Japanese population, the difference in posterior facial height and mandibular plane angle did not measure a significant difference [27]. Variations in the study could be due to the cohort studied, as the East Asian population has a straighter facial profile than other Asian populations.

Various studies have measured the vertical relationship of dentoalveolar compensation. However, no study has compared and correlated vertical and horizontal skeletal and dental compensation. Regarding the negative relationship between maxillary and mandibular inclination and vertical patterns, Kuitert et al. [28] observed stable results in both short- and long-face groups. Similarly, Modi et al. [16] identified a negative correlation between mandibular incisor inclination and vertical patterns, while no significant correlation was observed between maxillary incisor inclination and vertical patterns.

The SNA and SNB angles negatively correlate with LAFH, UAFH, PFH, and AFH. A smaller angle of SNB increases UAFH and LAFH lengths, which results in increased AFH length. A strong negative correlation (−0.225) between the SNA angle and the U1-NA and L1/MP values in class I malocclusion indicates that as the SNA angle increases, the U1-NA value decreases. A strong negative correlation of −0.542 exists between ANB, U1-NA, and U1/PP, indicating that as SNB decreases, there is a tendency for the mandible to rotate in a downward direction. The ANB angle, with a mean value of 7.6, exhibits a positive correlation with the S-Go and FH ratios. This suggests that as the ANB angle increases, there is a tendency for the mandible to rotate downward and backward. There is no association between ANB and AFH, LAFH, and UAFH. The ANB angle exhibits a negative correlation with PFH, suggesting that an increase in the ANB angle is associated with a decrease in PFH. Posterior height was weakly correlated with maxillary and mandibular length and positively correlated with their anteroposterior position. Schudy proposed that vertical and anteroposterior growth are opposing forces, which is a plausible explanation considering that anteroposterior facial height can be seen as vertical height in extremely long or short face types [29]. Several studies have indicated that in cases of relative normal overbite, the vertical growth of the PFH is in line with anteroposterior growth [8,10,29].

The management of vertical dimensions during orthodontic treatment is a challenging issue. Orthodontic treatment requires thorough knowledge regarding vertical measurements [17,22,28]. Vertical measurements include mainly AFH and PFH. This study found a favorable correlation between UAFH, LAFH, AFH, PFH, and the Jarabak ratio. This illustrates that the LAFH, AFH, PFH, and FH ratios are low when UAFH decreases. The correlation between UAFH and AFH is 0.767. LAFH positively correlates with AFH, PFH, F prop, and FH ratio, whereas UAFH does not. A significant correlation (0.856) was recorded between LAFH and AFH, indicating LAFH increases if AFH shows downward and forward growth in class I and II malocclusions.

In patients with a normal overbite and a forward rotation of the mandible, the vertical measurement does not depend on the PFH growth combined with a relatively short AFH. The mandibular forward rotation is not caused by a simultaneous increase in PFH and a decrease in AFH, but rather by significant increases in both dimensions [29]. The study findings indicate that the development of PFH, rather than AFH, is the primary factor influencing mandibular rotation. This aligns with Björk's statement that, in optimal conditions, the pivot point for anterior mandibular growth rotation is situated at the incisors [30]. Consequently, forward rotation increases the AFH and PFH. A face with a low angle, characterized by a long ramus and a wide frontal region (wide bizygomatic), can create the perception of a short face, even if the height of the anterior lower face is within the normal range. This phenomenon accounts for the tendency to perceive faces with a low angle as having a shorter appearance, while faces with a wider shape and a square profile contribute to the perception of a shorter face.

Numerous studies have emphasized the significance of AFH and LAFH values due to their substantial impact on developing vertical facial disproportions [17,24]. Facial height reduction can be achieved by examining mandibular molars for forward and backward rotation. This technique can be utilized in treating adolescent patients with skeletal and dental classes I, II, and II malocclusions resulting from the angle and growth of the ramus and body of the mandible. This association generally causes a lowered posterior facial height, a steeper mandibular plane angle, an increased ANB angle between SNA and SNB plane angles, a normal SNA angle with a decreased SNB angle, an increased angle of convexity, and an increased overjet.

Limitations and future recommendations

The major limitation of the current study is the limitation of subjects as this is a single-center study and the results could not be generalized. Future studies should be planned to evaluate the dental and skeletal parameters of Saudis residing in other provinces and compare them with the present study's findings. In addition, different types of measurement, including three-dimensional computed tomography, should be evaluated to record different skeletal and dental malocclusions among the Saudi population. Finally, the basic characteristics of different malocclusions needed to be evaluated among the Saudi people.

## Conclusions

Following the evaluation of these cephalometric findings, it could be concluded that horizontal and vertical measurements play a fundamental role in determining the alignment and position of teeth within the oral cavity. In dental malocclusion classes I and II, SNB angles were small, with the increase in UAFH and LAFH resulting in increased anterior facial height, indicating a positive association. In dental malocclusion class I, a strong positive relationship was recorded between ANB angle and LAFH; conversely, in dental malocclusion class III, a strong negative relationship was established between ANB and LAFH; these findings indicate a tendency for the mandible to rotate downward and backward. These measures also imply changes in skeletal parameters resulting in overjets and angulations of the anterior teeth. A significant correlation was measured between dentoalveolar and skeletal growth patterns in dental class I malocclusion among the Jazan population. Hence, understanding the relationship between vertical and horizontal skeletal growth patterns can help orthodontists modify the positioning of the teeth to correct skeletal discrepancies in the vertical relation, overall preventing excessive compensation. Understanding these relationships is crucial for achieving functional and aesthetic results in orthodontic treatment.

## Appendices

**Table 6:** Correlation between vertical and horizontal skeletal relation **Pearson correlation has a higher negative or positive relation between the two parameters. *Pearson correlation has a low negative or positive relation between the two parameters. Significance was p-value<0.05.

		AFH (N-Me)	PFH (S-Go)	UAFH (N-ANS)	LAFH (ANS-Me)	FH ratio SGo/Nme	F prop LAFH/Nme
A-NV	Correlation coefficient	−0.207^**^	−0.153^*^	−0.097	−0.197^**^	−0.038	−0.090
	Sig. (two-tailed)	0.001	0.013	0.114	0.001	0.531	0.143
B-NV	Correlation coefficient	−0.188^**^	−0.047	−0.083	−0.178^**^	0.071	−0.092
	Sig. (two-tailed)	0.002	0.444	0.175	0.003	0.249	0.134
Ab diff (NV)	Correlation coefficient	0.060	−0.093	0.047	0.049	−0.157^*^	0.022
	Sig. (two-tailed)	0.327	0.130	0.449	0.429	0.010	0.721
SNA	Correlation coefficient	−0.167^**^	−0.138^*^	−0.088	−0.155^*^	−0.053	−0.069
	Sig. (two-tailed)	0.006	0.024	0.154	0.011	0.389	0.263
SNB	Correlation coefficient	−0.123^*^	−0.025	−0.044	−0.126^*^	0.063	−0.077
	Sig. (two-tailed)	0.045	0.683	0.470	0.039	0.306	0.210
ANB	Correlation coefficient	−0.067	−0.150^*^	−0.016	−0.074	−0.136^*^	−0.043
	Sig. (two-tailed)	0.278	0.014	0.796	0.227	0.026	0.480
Skeletal Class	Correlation coefficient	0.012	-0.017	0.034	-0.012	−0.056	−0.066
	Sig. (two-tailed)	0.844	0.786	0.582	0.849	0.358	0.283

**Table 7:** Correlation between teeth inclination and vertical skeletal components **Pearson correlation has a higher negative or positive relation between the two parameters. *Pearson correlation has a low negative or positive relation between the two parameters. Significance was p-value<0.05.

		AFH (N-Me)	PFH (S-Go)	UAFH (N-ANS)	LAFH (ANS-Me)	FH ratio SGo/Nme	F prop LAFH/Nme
U1–NA	Correlation coefficient	0.227^**^	0.165^**^	-0.067	0.311^**^	0.042	0.297^**^
	Sig. (two-tailed)	0.000	0.007	0.279	0.000	0.489	0.000
L1-NB	Correlation coefficient	0.201^**^	0.014	−0.076	0.274^**^	−0.091	0.294^**^
	Sig. (two-tailed)	0.001	0.823	0.214	0.000	0.137	0.000
U1/PP	Correlation coefficient	−0.069	−0.046	−0.110	−0.045	−0.020	−0.025
	Sig. (two-tailed)	0.260	0.455	0.073	0.460	0.746	0.687
L1/MP	Correlation coefficient	−0.171^**^	0.066	−0.166^**^	−0.132^*^	0.294^**^	−0.033
	Sig. (two-tailed)	0.005	0.279	0.006	0.031	0.000	0.591
Dental Class	Correlation coefficient	0.023	−0.050	−0.028	0.050	−0.101	0.041
	Sig. (two-tailed)	0.712	0.420	0.654	0.418	0.099	0.504

**Table 8:** Correlation between teeth position and inclinations and dental class **Pearson correlation has a higher negative or positive relation between the two parameters. *Pearson correlation has a low negative or positive relation between the two parameters. The significance was p-value<0.05.

		U1–NA	L1-NB	U1/PP	L1/MP	Dental class
U1–NA	Correlation coefficient	1.000	0.133^*^	0.611^**^	0.011	0.044
	Sig. (two-tailed)		0.029	0.000	0.858	0.471
L1-NB	Correlation coefficient		1.000	0.183^**^	0.624^**^	−0.073
	Sig. (two-tailed)			0.003	0.000	0.234
U1/PP	Correlation coefficient			1.000	0.173^**^	0.046
	Sig. (two-tailed)				0.005	0.451
L1/MP	Correlation coefficient				1.000	−0.073
	Sig. (two-tailed)					0.235

**Table 9:** Correlation between different horizontal angles and teeth positions/ inclination anterior-posterior relation **Pearson correlation has a higher negative or positive relation between the two parameters. *Pearson correlation has a low negative or positive relation between the two parameters. Significance was p-value<0.05.

		U1–NA	L1-NB	U1/PP	L1/MP
A-NV	Correlation coefficient	−0.311^**^	0.300^**^	0.080	0.233^**^
	Sig. (two-tailed)	0.000	0.000	0.195	0.000
B-NV	Correlation coefficient	0.191^**^	−0.121^*^	0.432^**^	−0.069
	Sig. (two-tailed)	0.002	0.048	0.000	0.261
Ab diff (NV)	Correlation coefficient	−0.554^**^	0.422^**^	−0.510^**^	0.285^**^
	Sig. (two-tailed)	0.000	0.000	0.000	0.000
SNA	Correlation coefficient	−0.273^**^	0.286^**^	0.049	0.194^**^
	Sig. (two-tailed)	0.000	0.000	0.423	0.001
SNB	Correlation coefficient	0.241^**^	−0.092	0.473^**^	−0.065
	Sig. (two-tailed)	0.000	0.133	0.000	0.292
ANB	Correlation coefficient	−0.658^**^	0.490^**^	−0.417^**^	0.364^**^
	Sig. (two-tailed)	0.000	0.000	0.000	0.000
